# Reduced arterial elasticity after anabolic–androgenic steroid use in young adult males and mice

**DOI:** 10.1038/s41598-022-14065-5

**Published:** 2022-06-11

**Authors:** H. S. Melsom, C. M. Heiestad, E. Eftestøl, M. K. Torp, K. Gundersen, A. K. Bjørnebekk, P. M. Thorsby, K. O. Stensløkken, J. Hisdal

**Affiliations:** 1 Institute of Clinical Medicine, Faculty of Medicine, University of Oslo, Oslo, Norway; 2 Department of Vascular Surgery, Oslo University Hospital, Oslo, Norway; 3 Institute of Basic Medical Sciences, Faculty of Medicine, University of Oslo, Oslo, Norway; 4 Department of Biosciences, Faculty of Mathematics and Natural Sciences, University of Oslo, Oslo, Norway; 5 The Anabolic Androgenic Steroid Research Group, Oslo University Hospital, Oslo, Norway; 6 Biochemical Endocrinology and Metabolism Research Group, Hormone Laboratory, Department of Medical Biochemistry, Oslo University Hospital, Oslo, Norway

**Keywords:** Cardiovascular biology, Risk factors, Vascular diseases, Arterial stiffening, Carotid artery disease

## Abstract

High-doses of anabolic–androgenic steroids (AAS) is efficient for building muscle mass, but pose a risk of cardiovascular side effects. Little is known of the effect of AAS on vasculature, but previous findings suggest unfavorable alterations in vessel walls and vasoreactivity. Here, long-term effect of AAS on vascular function and morphology were examined in male weightlifters, and in a mimicking animal model. Arterial elasticity and morphology were tested with ultrasound, pulse wave velocity (PWV) and carotid intima media thickness (cIMT) in 56 current male AAS users, and 67 non-exposed weightlifting controls (WLC). Female mice were treated with testosterone for 14 days and echocardiography were applied to evaluate vascular function and morphology. Male AAS users had higher PWV (p = 0.044), reduced carotid artery compliance (p = 0.0005), and increased cIMT (p = 0.041) compared to WLC. Similar functional changes were found in the ascending aorta of mice after 7- (p = 0.043) and 14 days (p = 0.001) of testosterone treatment. This animal model can be used to map molecular mechanisms responsible for complications related to AAS misuse. Considering the age-independent stiffening of major arteries and the predictive power of an increase in PWV and cIMT, the long-term users of AAS are at increased risk of severe cardiovascular events.

## Introduction

Anabolic–androgenic steroids (AAS) are a family of compounds that includes the male sex hormone testosterone, and its synthetic derivatives^1,2^. The recognition of the anabolic properties of AAS led to AAS use propagating into elite sports in the 1950s, where the use of androgens expanded during the next decade before being banned^3^. AAS abuse are no longer reserved for elite athletes but are now commonly used by non-competitive athletes and bodybuilders^4–7^.

AAS use has been associated with a range of adverse medical and psychological consequences^5^. Growing evidence links high dose AAS use to a variety of cardiovascular complications, including arterial stiffness. Studies have demonstrated that administration of supraphysiological doses of AAS can potentially lead to structural alterations of the vessel walls and cause endothelial dysfunction that can affect the elastic properties of the vasculature, induce formation of plaque and be a precursor in development of atherosclerosis^8,9^. The elasticity in large arteries contributes to reduced fluctuations in blood pressure within the cardiac cycle and ensure a steady and continuous blood flow to the peripheral vasculature^10^. Loss of arterial elasticity is mainly a result of overproduction of collagen^11^. Arterial stiffening is one of the major cardiovascular disease risk factors, often found in association with aging^12^. Loss of arterial elasticity in the larger central arteries, contributes greatly to the development of heart disease related to systolic hypertension^10,13^. Carotid femoral pulse wave velocity (PWV) is a non-invasive and robust method that has emerged as the gold standard for assessment of arterial stiffness^14^. PWV increases with age and in the presence of other cardiovascular risk factors. It is used as a direct measure of arterial stiffness^15^.

Unfortunately, a variety of confounding factors in many studies makes it hard to estimate the true negative health-related effects from AAS use. Most conclusions are based on small heterogeneous populations with diverse usage patterns or forensic studies, where the physical phenotype after death suggest AAS misuse as major cause^16,17^. History of use is often self-documented and the majority of the participants are using a mixture of different doses and types of AAS^16,18^. More cross-sectional and longitudinal studies are therefore necessary to evaluate the true physiological effects of AAS use. Due to ethical and legal reasons, studies under such controlled conditions are difficult or not possible to implement. In the present study, we have studied men that have reported minimum 1 year of accumulated AAS exposure within the last 12 months and compared them with men with no experience with AAS. The main aim of the study was to investigate the long-term effect of AAS use on vascular function and morphology in young users.

Based on previous studies, we hypothesized that high-dose administration of AAS would lead to unfavorable vascular alterations. We also aimed to establish an animal model for further studies of mechanistic changes caused by high-dose AAS exposure.

## Results

### Anabolic–androgenic steroid users and weightlifting controls

#### Demographical data

Table 1 shows demographical data for AAS users and weight lifting controls (WLC). There was no difference between the two groups with respect to age, height, systolic blood pressure or training hours per week. The AAS users had significantly higher body weight and lifted more than the WLC. The AAS users did also have significantly lower education than the WLC. Cholesterol- and hormone levels are presented in supplemental Table 1.

**Table 1:** Demographical data for the study subjects.

	AAS users (n = 56)	WLC (n = 67)	p-value
	Median (25th–75th percentile)	Median (25th–75th percentile)	
Age, years	39 (32–49)	38 (30–45)	0.389
Height, m	1.8 (1.8–1.9)	1.8 (1.8–1.9)	0.074
Weight, kg	100 (94–111)	93 (84–100)	< 0.001*
BMI, kg/m^2^	30.7 (28.7–33.1)	27.7 (26.2–30.7)	< 0.001*
SBP, mmHg	130 (120–140)	125 (120–130)	0.060
DBP, mmHg	80 (80–85)	80 (75–85)	0.022*
MAP, mmHg	97 (93–103)	93 (90–100)	0.013*
AAS debut age, years	20 (18–24)	–	–
Weekly dose of AAS, mg	750.0 (415.0–1262.5)	–	–
Accumulated time of use, years	11.0 (6.5–17.5)	–	–
Education, years	15 (13–16)	17 (15–19)	< 0.001*
Weight training, hours per week	5.7 (4.0–9.3)	5.9 (3.9–8.0)	0.789
Endurance training, hours per week	0.7 (0.0–2.0)	1.0 (0.2–2.0)	0.489
Bench press, kg	175.0 (152.5–206.0)	140.0 (134.0–159.0)	< 0.001*
Ground lift, kg	240.0 (210.0–275.0)	200.0 (180.0–240.)	< 0.001*
Alcohol, yes	34	56	0.004*
Alcohol, units per week, median (25th–75th percentile)	2 (0–4)	2 (0–5)	0.406
Smoke, yes	4	0	0.022
Other tobacco, yes	15	17	0.700

*AAS* anabolic androgenic steroids, *WLC* weight lifting controls, *BMI* body mass index, *SBP* systolic blood pressure, *DBP* diastolic blood pressure, *MAP* mean arterial pressure. *Significant difference between groups.

#### Vascular function

**Figure 1:**
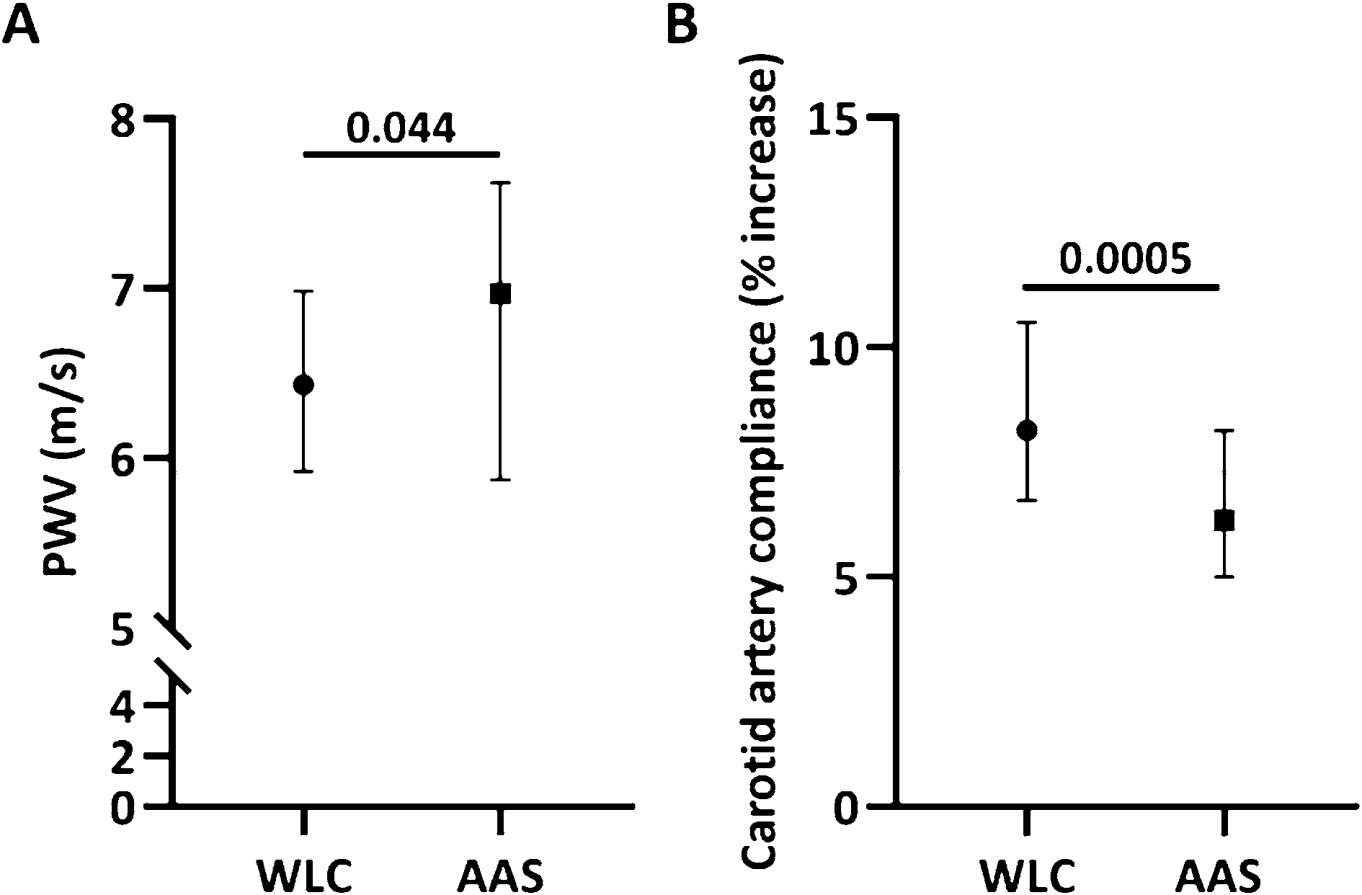
Vascular function: (**A**) Pulse wave velocity (PWV) in anabolic androgenic steroids (AAS) users (N = 56) and weight lifting controls (WLC) (N = 67). Data presented as median with interquartile range. (**B**) Carotid artery compliance in AAS users (N = 56), compared to WLC (N = 58). Percentage increase calculated from the difference in inner diameter (mm) of the left carotid artery within one heart cycle. Data presented as median with interquartile range.

AAS users had higher PWV compared to the WLC, when adjusted for age [6.97 m/s (5.87–7.62 m/s), 6.44 m/s (5.92–6.99 m/s) respectively, p = 0.044] (Fig. 1A). Reduced carotid artery compliance was also seen among the group of AAS users, when compared to the WLC and adjusted for age [6.24% (5.00–8.18%), 8.19% (6.67–10.55%) respectively, p = 0.0005] (Fig. 1B).

#### Vascular morphology

**Figure 2:**
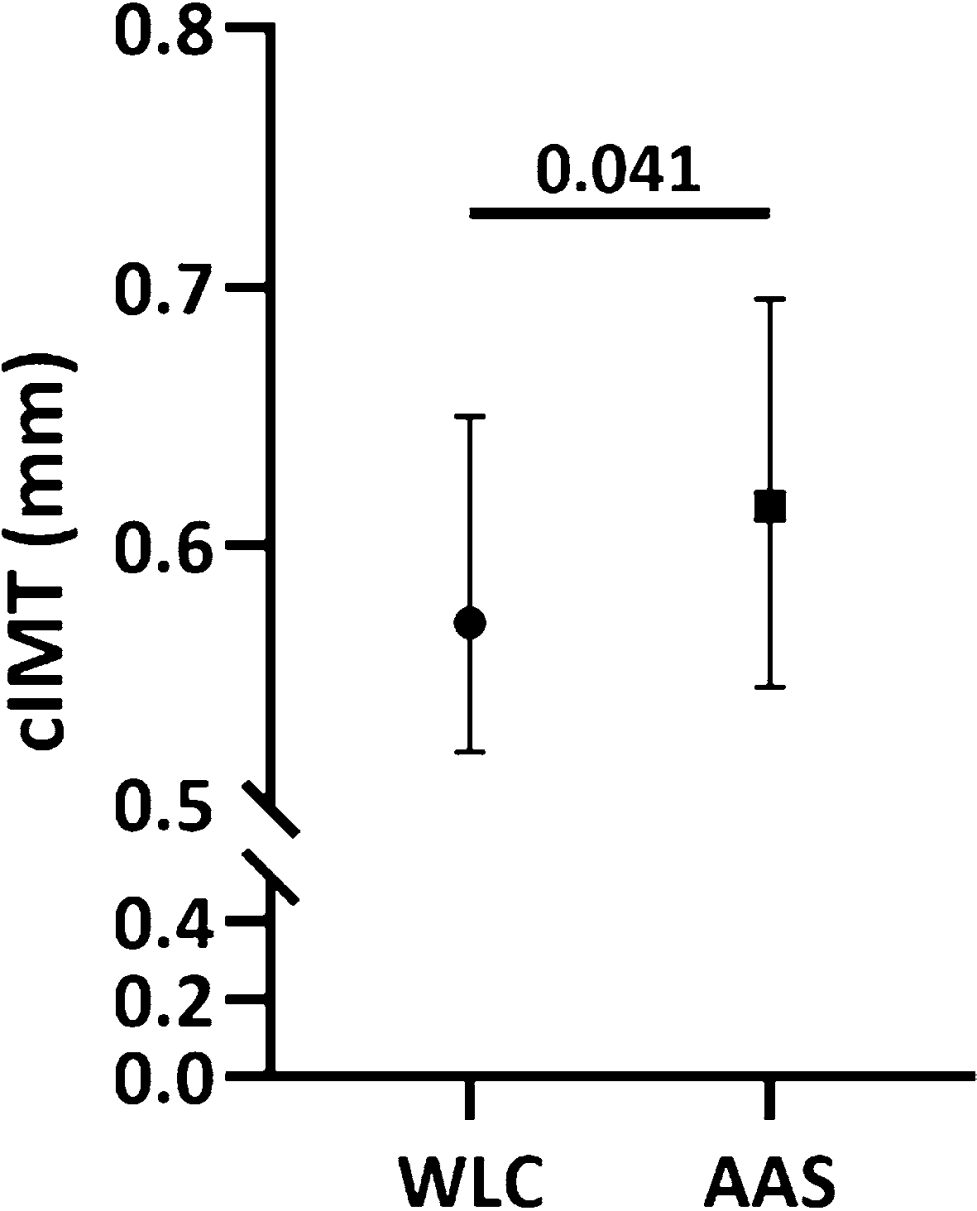
Vascular morphology: Carotid intima-media thickness (cIMT) (mm) in the group of anabolic–androgenic steroid (AAS) users (N = 56) compared to the WLC (N = 67). Data presented as median with interquartile range.

Thickness of intima media of the common carotid artery (cIMT) was measured in both groups and thickening of the cIMT is an early sign of arteriosclerosis. AAS users had significantly thicker cIMT than the WLC [AAS users; 0.62 mm (0.55–0.70 mm), WLC; 0.57 mm (0.52–0.65 mm), p = 0.041] (Fig. 2).

#### Substance use

**Figure 3:**
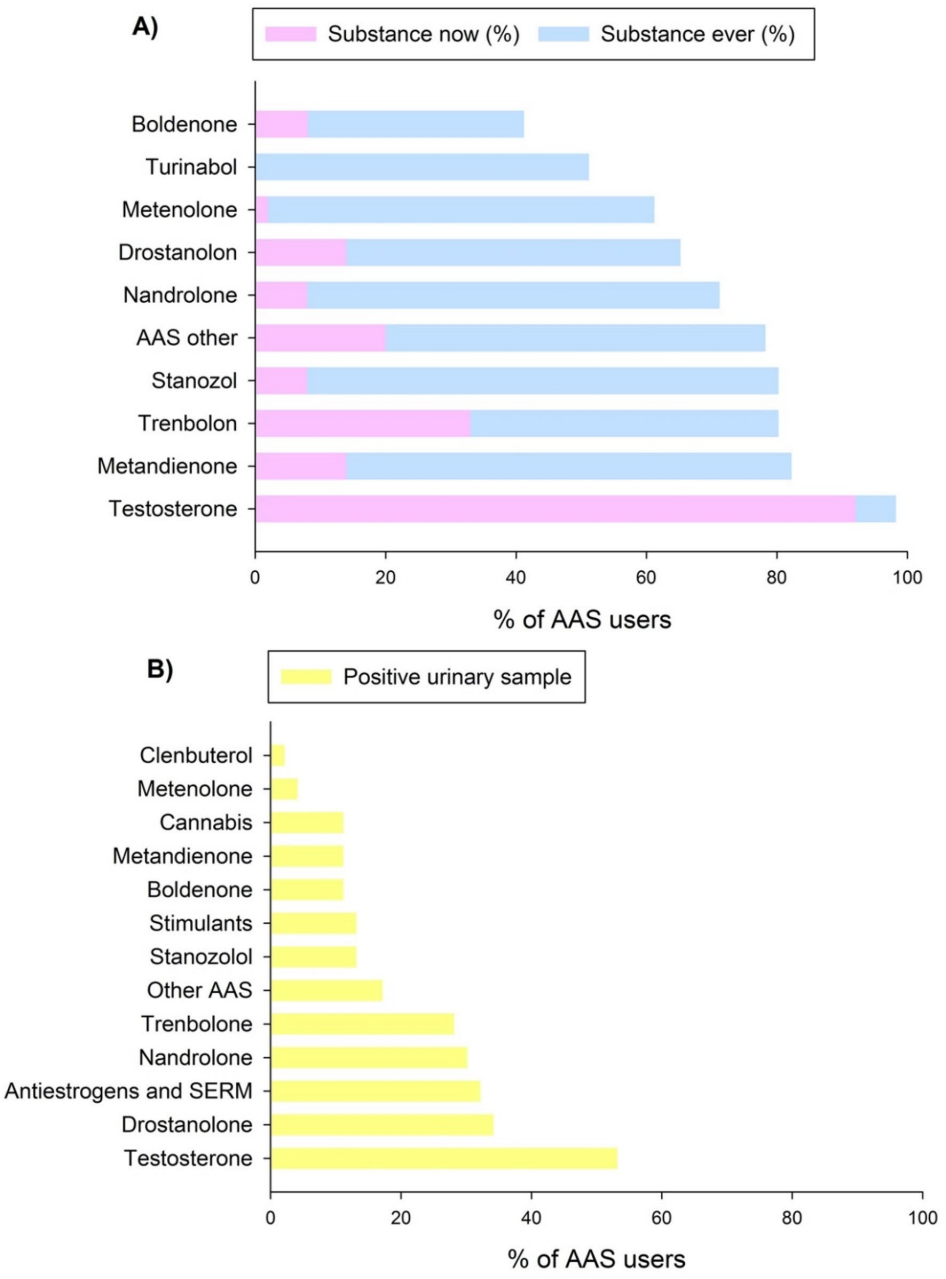
Substance use: (**A**) Substances reported by the anabolic–androgenic steroid (AAS) users (N = 50). Substance preferred is the substance used now. Substance ever is all substances ever used. (**B**) Substances the AAS users (N = 50) tested positive for in the urine samples.

Figure 3A shows which substances the AAS users reported and Fig. 3B shows which substances was detected in urine samples.

### Animal experiments

#### Vascular function

**Figure 4:**
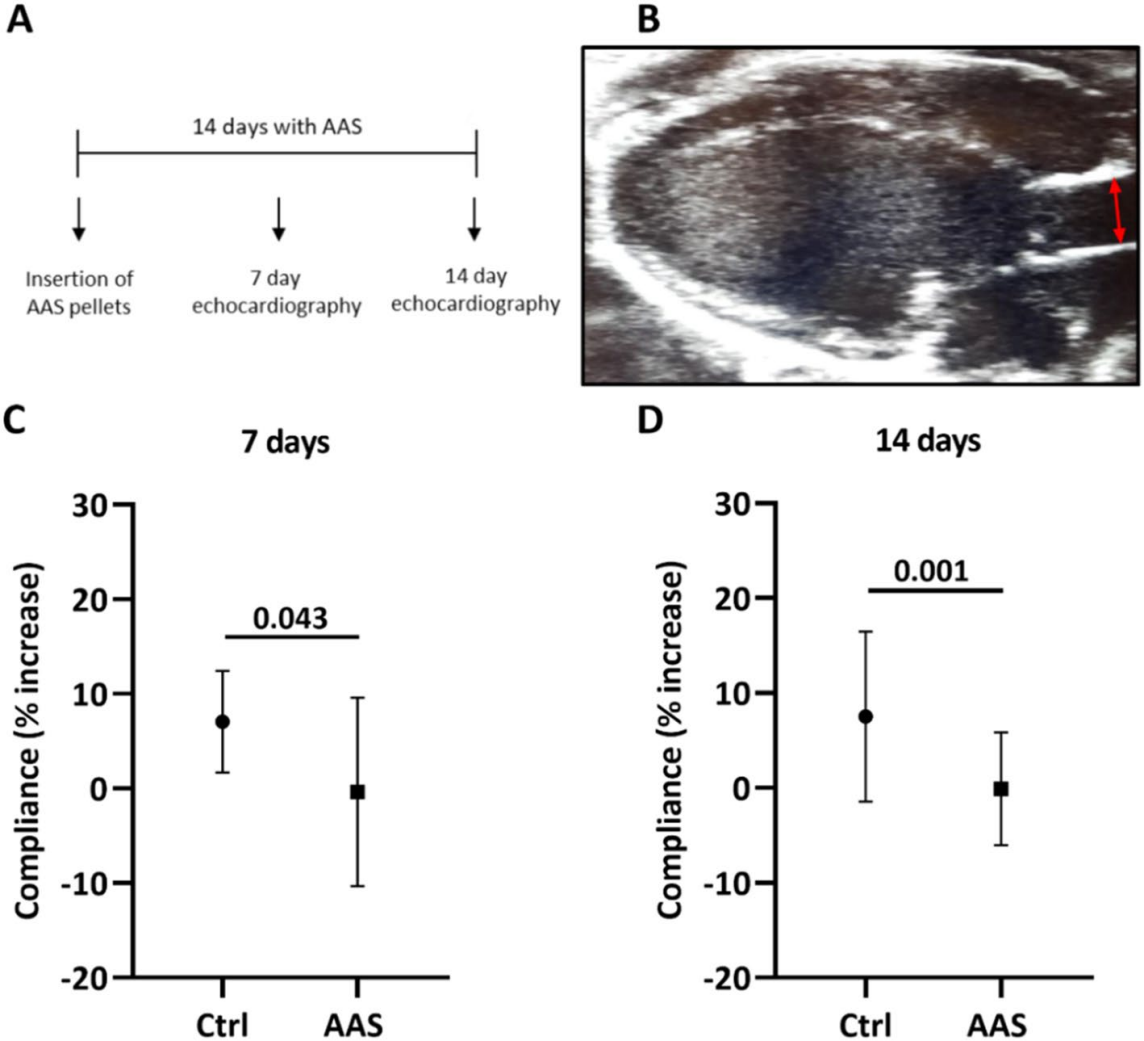
Method description and vascular function: (**A**) Timeline describing the series of experimental events in the animal study. Animals were exposed to testosterone (AAS) or sham pellets for 14 days. (**B**) B-mode image of a mouse heart acquired from 2D echocardiography showing the left ventricle in a parasternal long axis view, apex pointing to the left and aorta to the right. Double-headed arrow illustrates area of diastolic- and systolic diameter measurements of the ascending aorta. (**C**,**D**) Aortic compliance after 7 and 14 days of testosterone treatment, measured as percentage increase calculated from the difference in inner diameter (mm) of the ascending aorta within one heart cycle. Testosterone treated animals = AAS (N = 15), sham operated animals = Ctrl (N = 20). Data presented as mean ± SD.

After exposure to high doses of testosterone, female mice had reduced arterial compliance. After 7 days, compliance of the ascending aorta decreased in testosterone treated animals (− 0.35% ± 9.97%) compared to control animals (7.07% ± 5.39%), p = 0.042 (Fig. 4C). Elasticity decreased even further after 14 days (− 0.09% ± 5.93) (7.52% ± 8.97), p = 0.001 (Fig. 4D).

#### Vascular morphology

**Figure 5:**
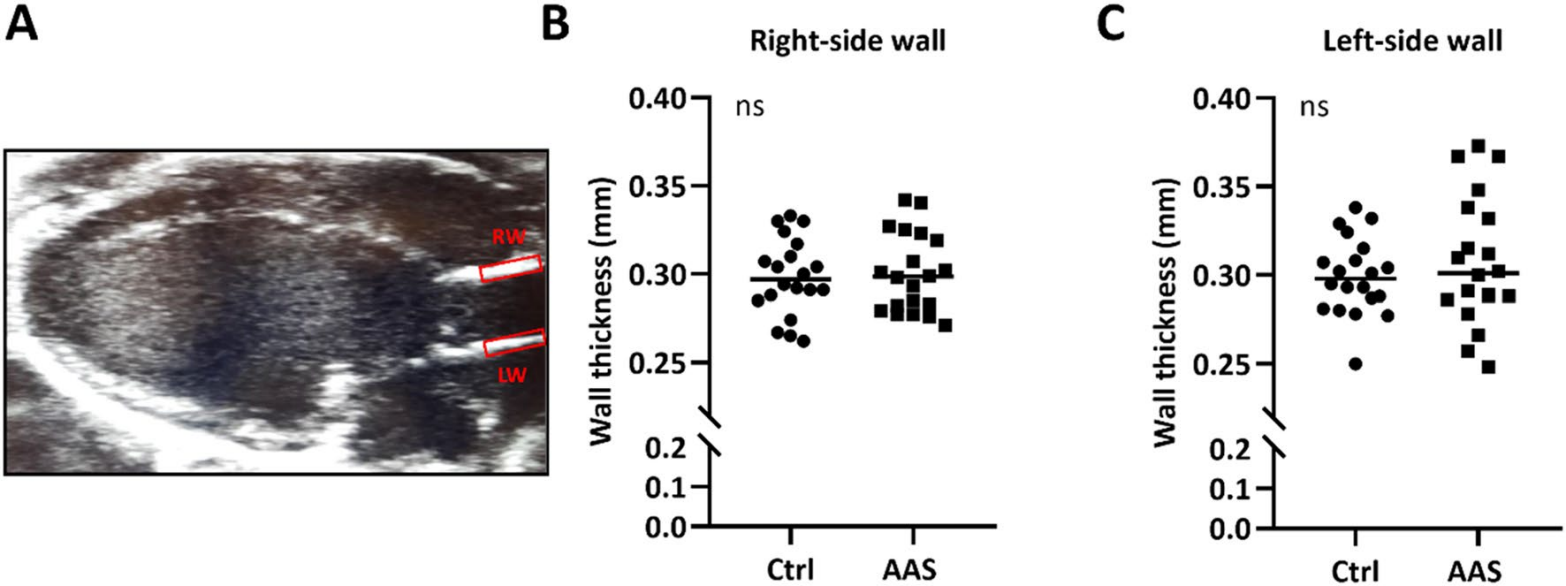
Vascular morphology: (**A**) B-mode image of a mouse heart acquired from 2D echocardiography showing the left ventricle in a parasternal long axis view, apex pointing to the left and aorta to the right. The boxes illustrates the areas of the ascending aorta used for measurements of wall thickness. *RW* right-side wall, *LW* left-side wall. (**B**,**C**) Wall thickness measured on the RW and LW of the ascending aorta after 14 days of testosterone exposure. Testosterone treated animals = AAS (N = 20), sham operated animals = Ctrl (N = 20). Data presented with median.

Wall thickness of the ascending aorta was measured in mice. After 14 days of testosterone exposure, wall thickness did not differ on either the right- (Fig. 5B) or the left-side (Fig. 5C) of the ascending aorta, compared to controls.

## Discussion

Accumulating evidence suggests that prolonged AAS use may harm the vascular morphology and function, but large-scale longitudinal studies with proper controlled conditions are lacking. By combining animal experiments and blood vessel examination of long-term AAS users and WLC, we found evidence of unfavorable changes in the vasculature caused by high-dose AAS use. AAS users had thicker cIMT, higher PWV, and decreased artery compliance, compared to WLC. Similar findings were seen with mouse echocardiography, where loss of aortic compliance of the ascending aorta was found after only few days of testosterone treatment.

Androgens have great impact and broad effects on the heart and vasculature. Natural age-related testosterone depression and testosterone deficiency have negative impact on general health and is associated with increased risk of cardiovascular complications and mortality^19^. However, there is controversy regarding the testosterone supplements used to compensate for age-related depression. While some studies claim the effect of testosterone is exclusively beneficial to the cardiovascular system, there are also reports of harmful effects^20^. However, AAS administered at supraphysiological doses produce a large range of adverse cardiovascular effects, including atherosclerosis and hypertension^5,21^.

Thickening of the cIMT is an early predictive morphological marker for future cardiovascular and cerebrovascular events^22^. Thickening of cIMT is common among the elderly and is linearly associated with rising age^22^. While common age-specific reference values for cIMT thickness is difficult to determine, thickening of cIMT is a precursor of atherosclerosis^23^. Even though the participants in the present study are relatively young and perform strength training regularly, the AAS group had a significantly higher cIMT than the WLC, and in the upper limit of the normal reference range within the sub-population of healthy men^23^.

It has been shown that long-term high-dose AAS administration could have an unfavorable effect on endothelium and smooth muscle dependent vascular responses in humans^9^. We found that PWV was significantly higher among the AAS users compared to the WLC. PWV has been shown to be an important parameter when assessing potential cardiovascular risk, independently of traditional risk factors and in diversified populations^24^. There is evidence that arterial stiffness, cIMT, plaques in the carotid arteries, and presence of peripheral arterial disease are highly correlated. All of them are known risk factors for cardiovascular events^25^. An association between stiffening of the main arteries and mortality has been found in studies of patients with underlying diseases affecting the vasculature, such as hypertension or end-stage renal disease^24,26^. Hence, the significantly higher PWV observed among young AAS users is worrying.

In the present study, we observed functional changes in central parts of the vasculature in both humans and mice after administration of androgens. Users of AAS had significantly reduced carotid compliance compared to non-users. These results are in accordance with our observations from the animal experiments. Mice exposed to high-dose testosterone had significantly decreased compliance in the ascending aorta, compared to sham animals. Female mice were chosen due to stable, low circulating levels of testosterone, compared to male mice^27^. Reduced compliance were detected already after 7 days of treatment. Unfavorable aortic alterations after use of AAS have previously been shown^21^. In a study with small sample size, male bodybuilders using AAS had decreased aortic distensibility compared to non-users^28^. Alterations in the visco-elastic properties of the wall leads to earlier arrival of the reflected wave in aorta during diastole, which increases cardiac workload and affects coronary blood flow^29,30^. Previous studies have shown that there is a link between the use of AAS and increased blood pressure and progression towards hypertension^21,31,32^.

Similarly, we find an increase in mean arterial pressure in AAS user compare to WLC. Unfortunately, we were not able to measure blood pressure non-invasively in mice. Elevated blood pressure have been shown in high-dose testosterone treated mice before^33^, thus it is unlikely that decreased arterial compliance measured here, is a result of reduced blood pressure. Even though the relationship between changes in the elastic properties of aorta and wall thickness is not defined, association between hypertension and atherosclerotic plaque formation, decreased aortic distensibility and wall thickening have been demonstrated^34,35^.

A few studies have examined cardiac effects of AAS exposure in animal models. Rats exposed to high-doses of AAS at young age developed permanent damage to the heart^36^. In addition to being nephrotoxic, long-term exposure have been shown to have detrimental effects on rabbit hearts, such as myocardial tissue damage and impaired diastolic function^37,38^. In the present study, the primary treatment was high doses of testosterone. However, it is possible that the introduction of testosterone would affect the endocrine system and thus alter the levels of other hormones, which may have a contributing or decisive effect on the observed vascular changes. The high incidents of acute myocardial infarction^39,40^ and sudden cardiac death in young users of AAS^41–43^, should sharpen our attention around the use of these types of substances. In order to form a clearer picture of the true physiological and molecular effects of AAS, animal studies with well-established models will be essential.

All substances reported by the AAS users were not detected in the urine. This can be explained by the variation in elimination half-life and detection times. Half time for the different substances varies from hours to weeks and the detection time in urine differ for days to months. Therefore, not all reported substances were detected. The blood test indicated current or resent use of AAS, since all had decreased gonadotrophins and SHBG and increased free adrenal index (FAI), compared to the WLC group.

Long-term use of AAS may lead to increased cIMT, increased PWV and decreased arterial elasticity. These findings were supported by functional vascular alterations after exposure of supraphysiological doses of testosterone in young mice. The results of the present study indicate that long-term use of AAS is a major threat to cardiovascular health, with increased risk of developing severe cardiovascular events, such as myocardial infarction and stroke.

## Materials and methods

### Ethical approvals

All experiments were approved by the Regional Committees for Medical and Health Research Ethics South East Norway (REC) (2013/601), and the research was carried out in accordance with the Declaration of Helsinki. All participants received oral and written information prior to participation, and gave written informed consent. The participants were compensated for their participation with a gift certificate equivalent to 500 NOK (approximately 60 USD). Experiments including animals were approved (FOTS ID 17736/25406) and performed in adherence with the Norwegian Animal Health Authority, Mattilsynet and the ARRIVE guidelines.

### Human participants

#### Study population

In total, 123 men engaged in heavy resistance strength training were included in the study. Participants were recruited through social media, posts on internet forums for bodybuilders, strongman, fitness, and weightlifting, and both open and closed forums directly targeting AAS users. Posters and flyers were also distributed in gyms in Oslo. In the ads, we called for men that had managed to bench press 120 kg (~ 265 pounds) for at least one repetition, and report ≥ 1 year of accumulated AAS exposure within the last 12 months or no experience with AAS. In the latter group, 100 kg (~ 220 pounds) bench press was the minimum criterium for inclusion. During recrutation, participants were offered an examination of the vasculature. All participants agreeing to this was included. Participants were divided into two groups: AAS users (n = 56, 49 currently on AAS) and weight lifting controls (WLC) with no history of AAS use (n = 67). To confirm alleged AAS use, all participants were tested as described by Bjørnebekk et al.^44^, with urine samples, questionnaire and interviews. As previously described, doping analysis confirmed the use of AAS among current users and confirmed no use among WLC^45^. Demographical data for the participants included in the final analysis are presented in Table 1.

#### Laboratory analyses

Blood and urine samples were obtained at Department of Cardiology. Laboratory measures of total cholesterol, low-density lipoprotein (LDL) and high-density lipoprotein (HDL) were analysed at Department of Medical Biochemistry, Oslo University Hospital at the routine laboratory. The analyses follicle-stimulating hormone (FSH), luteinizing hormone (LH), sex hormone binding globulin (SHBG) all with Simens, Immulite. Total testosterone (TT) (Hormone Laboratory, LC-MSMS), free androgen index (FAI was calculated automatically by the following formula: testosterone (nmol/l) × 10/SHBG (nmol/l)). All hormones were analysed at the Hormone Laboratory, Oslo University Hospital. All reference ranges are according to laboratory standards of Oslo University Hospital. The analyses at the Department of Medical Biochemistry, Oslo University Hospital and the Hormone Laboratory, Oslo University Hospital, were all accredited according to ISO 15189 and ISO 17025 respectively.

#### Preparation

Subjects were fasted for > 6 h, refrained from exercise for ≥ 24 h and abstained from caffeine, tobacco, alcohol and vitamin C ≥ 18 h before testing. Before the measurements, subjects rested in a supine position for 10 min in a dark, quiet, temperature-controlled room (20 °C) followed by assessment of blood pressure, measured manually with pressure cuff and stethoscope.

#### Measurement of arterial stiffness (PWV)

To determine carotid femoral pulse wave velocity (cfPWV), pulse waveforms were recorded trancutaneously at the right carotid and right femoral artery, using the Sphygmocor apparatus (Atcor, West Ryde, Australia). The apparatus equalizes the arterial circumferential pressure to obtain accurate pressure waveforms, by applanation tonometry. Several recordings are made in each patient and the recordings considered to have the highest quality according to predetermined requirements [AtCor Medical, technical notes, http://atcormedical.com/technicalnotes.html) (accessed June 2014)] are selected for further analysis. The PWV analyses were performed according to expert consensus statements^46^. Briefly, pulse wave transit time from the heart to the two arteries was calculated by relating the R-wave in simultaneously recorded ECG to the up-stroke of the pulse wave at both the carotid and the femoral artery. The distances covered by the waves, were assimilated to the surface distance between the two recording sites, obtained by a measuring tape. The cfPWV was then calculated by the Sphygmocor apparatus as the transit time from the heart to either the carotid or the femoral artery and the distances from the carotid and femoral recording sites to the sternal notch: cfPW = Δ distance (meters)/Δ t (seconds).

#### Intima-media thickness (cIMT)

Intima media thickness was measured in a supine position on the left carotid artery. The carotid artery was imaged over a distance of approximately 2 cm, 1–2 cm proximal to bulbus, and cIMT was measured in an area free from plaque. Images were acquired using a L) linear probe on a Vivid E95 ultrasound machine (GE Healthcare, Chicago, US). Wall thickness was measured with semiautomatic edge-detection in the EchoPac Post-processing package (GE Healthcare, Chicago, US).

#### Elasticity of the left carotid artery

Inner diameter of the left carotid artery was measured during diastole and systole within the same cardiac cycle by blinded assessment. Arterial compliance was calculated as percentage increase in diameter of the lumen from diastole to systole.

### Animal experiments

#### Animals

C57BL/6JRj (Janvier Labs, Le Genest-Saint-Isle, France) female mice. The animals were kept in a controlled environment (12:12 h light:dark cycle, 23 °C, 55–60% humidity) with free access to water and food (ssniff-Spezialdiäten GmbH, Soest, Germany). All animals were acclimatized for one week prior to experiments.

#### Implantation of testosterone pellets

Mice (18.6 g ± 0.7 g) were anesthetized (2–3% isoflurane (N01A B06 (Isoflurane), Baxter AS, Norway) and placed on a heated platform (Kent Scientific, Torrington, US). Hair was removed from an area in the neck of the animal. Three pellets containing testosterone (“T-M—testosterone 30 days”) or sham pellets (“T-M—Placebo”) (Belma Technologies, Liège, Belgium) were implanted subcutaneously by making an incision in the skin of the neck, placing them side-by-side towards the posterior-lateral part of the back. Vetbond tissue adhevise (3 M, Minnesota, US) was used to close the incision.

#### Mouse echocardiography

Mice were anesthetized (1.5–3% isoflurane (N01A B06 (Isoflurane), Baxter AS, Norway) and placed on a heated imaging platform (Vevo 3100, VisualSonics, Toronto, Canada). Body temperature, heart- and respiration rate were constantly monitored. Mouse hearts were imaged using a frequency (40 MHz) linear array transducer (MX550D, VisualSonics, Toronto, Canada) on an echocardiography machine (Vevo 3100, VisualSonics, Toronto, Canada). All echocardiography acquisitions and image analyses were performed and evaluated by blinded assessment. VevoLAB analysis software was used for the analysis (VisualSonics, Toronto, Canada).

#### Aortic compliance and wall thickness

Images were obtained with 2D echocardiography of the beating heart after 7- and 14 days of testosterone treatment (Fig. 4A). Inner diameter of the ascending aorta and aortic wall thickness was measured in B-mode parasternal long-axis (PSLAX) view during diastole and systole within the same cardiac cycle (Figs. 4B, 5A). Aortic compliance was calculated as percentage increase in diameter of the lumen from diastole to systole.

#### Statistics

Statistical analyses were performed using GraphPad prism 8 (GraphPad Software, San Diego, CA, US) and SigmaPlot 14.5 (Systat Software, San Jose, CA). All data were normality tested with Shapiro–Wilk normality test. Mann–Whitney U Test was used to test for statistical differences between independent groups. PWV and carotid artery compliance were adjusted for age. Data are presented as median with interquartile range, unless otherwise is described. p-values below 0.05 were considered significant.

### Limitations

Some limitations should be considered when interpreting the results from this study. Firstly, this is a cross-sectional study, which does not allow for interpreting causality. However, results from the animal study supports the results from the human study. Second, in Norway, misuse of AAS is associated with health problems and are forbidden by law. Secrecy surrounding personal use makes it challenging to recruit users of AAS and thus study the effects. Diversity among choice of substances within the groups of AAS, polypharmacy, how they are consumed, as well as the duration of use can affect the vascular system differently. Thirdly, lifestyle factors, such as diet and drug use, are difficult to control for and might influence our results. This study only included men with Scandinavian ethnicity, and hence the results cannot be generalized to women or other ethnicities. For ethical reasons, it is not possible to design a study where one group receives a given amount and type of AAS. Consequently, we have to study individuals who are consuming AAS at their own initiative. Even though AAS users and WLC in the present study share the same interest in heavy resistance training and they are matched on training hours per week, discrepancy in exercise regimen can also affect the cardiovascular system differently.

Compliance was calculated from 2D mouse echocardiography images acquired in PSLAX view. These data are presented as percentage increase from diastole to systole within the same cardiac cycle. Normally, the diameter will increase in the ascending aorta just after ventricular systole. For some of the animals, the result of these calculations had a negative value. Due to the rigid artery walls of this experimental group, diameter change within the same cardiac cycle is minimal or not measurable. In addition to the technically challenging mouse echocardiography, the diameter was measured by drawing a line with the cursor manually in the analysis program. Minor measurement errors have therefore led to negative values.

## Supplementary Information

Supplementary Table 1.
