# Supplementary material for: Reduced arterial elasticity after anabolic–androgenic steroid use in young adult males and mice
Source: Sci Rep. 2022 Jun 11;12:9707. doi: 10.1038/s41598-022-14065-5 (PMC9188580; doi:10.1038/s41598-022-14065-5)
Supplement: Supplementary file 1 — Supplementary Table 1. [file 41598_2022_14065_MOESM1_ESM.pdf]

Supplementary information

Supplemental Table 1: Cholesterol- and hormone levels. AAS = anabolic androgenic steroids, WLC = weight lifting controls. * Significant difference between groups.

	AAS users Median (25th-75th percentile)	WLC Median (25th-75th percentile)	p-value
**Hb, mg/dL**	16.5 (15.6-17.7)		
**Total cholesterol, mmol/L**	4.3 (3.7-5.1)		
**LDL-cholesterol, mmol/L**	2.9 (2.3-3.9)		
**HDL-cholesterol, mmol/L**	0.9 (0.7-1.1)		
**Total cholesterol/HDL ratio**	5.0 (3.7-6.3)		
**HDL/LDL ratio**	0.3 (0.2-0.4)		
**Testosterone, nmol/L**	27.0 (10.8-47.5)	17.8 (13.0-21.3)	0.015*
**SHBG**	16.0 (8.0-33.5)	37.1 (28.4-46.2)	<0.001*
**Free testosterone ((testosterone x 10)/SHBG)**	12.5 (4.2-44.6)	4.8 (3.9-5.7)	<0.001*
**FSH, U/L**	0.1 (0.1-1.0)	4.5 (3.1-6.6)	<0.001*
**LH, U/L**	0.1 (0.1-0.3)	4.2 (3.5-5.3)	<0.001*
**T/E ratio**	30.4 (4.6-83.7)	0.8 (0.5-1.6)	<0.001*
